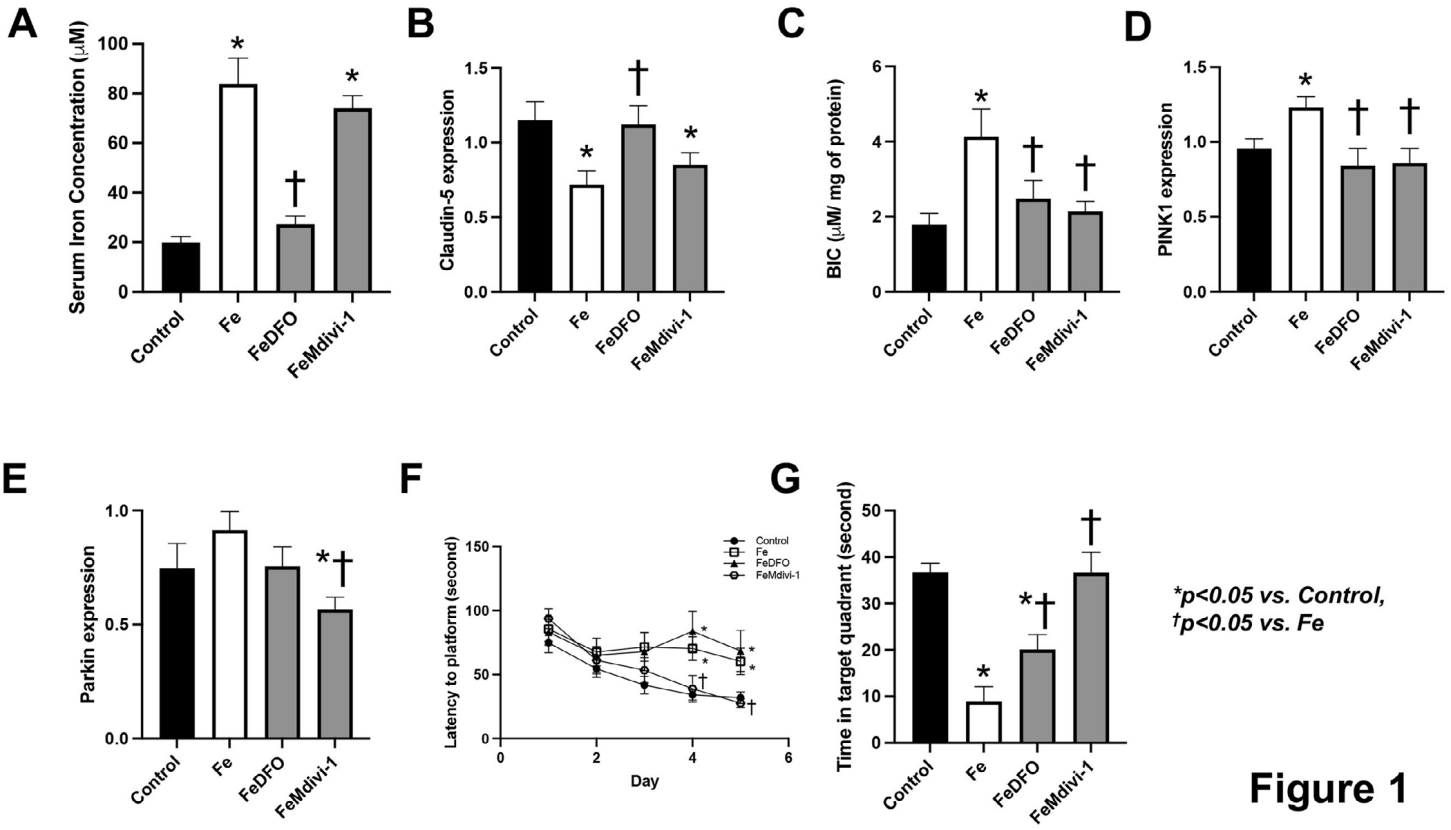# Mdivi‐1 Mitigated Excessive Brain Mitochondrial Fission and Brain Mitophagy, Consequently Restoring Spatial Memory in Rats Under Iron‐overloaded Condition

**DOI:** 10.1002/alz.085025

**Published:** 2025-01-03

**Authors:** Jirapas Sripetchwandee, Aphisek Kongkaew, Sirinart Kumfu, Nipon Chattipakorn, Siriporn C Chattipakorn

**Affiliations:** ^1^ Chiang Mai University/Neurophysiology Unit/Cardiac Electrophysiology Research and Training Center, Faculty of Medicine, Chiang Mai Thailand; ^2^ Chiang Mai University/Department of Physiology/Faculty of Medicine, Chiang Mai Thailand; ^3^ Chiang Mai University/Research Administration Section/Faculty of Medicine, Chiang Mai Thailand; ^4^ Chiang Mai University/Department of Oral Biology and Diagnostic Sciences/Faculty of Dentistry, Chiang Mai Thailand

## Abstract

**Background:**

Our studies suggest that iron‐overloaded rats developed neurotoxicity and cognitive impairment (1,2). An increase in brain mitochondrial fission and brain mitophagy have been considered as one of underlying mechanisms in brain with iron‐overloaded condition (3,4). Hence, a pharmacological intervention focused on preventing brain mitochondrial pathologies is required. The mitochondrial fission inhibitor, Mdivi‐1, has recently gained attention because of its beneficial effects on several neuropathological conditions including traumatic brain injury and intracerebral hemorrhage (5,6). However, the effectiveness of Mdivi‐1, particularly regarding brain mitochondrial parameters and cognitive function in iron‐overloaded condition, has not been investigated.

**Methods:**

Twenty‐four male adult Wistar rats were divided into 2 groups to receive an intraperitoneal injection with 10% dextrose in normal saline solution (NSS) as a control group (n = 6) or 100 mg/kg iron dextran as an iron‐overloaded group (n = 18). After 4 weeks of injection, the iron‐overloaded group was subdivided into 3 subgroups (n = 6/subgroup) to subcutaneously receive pharmacological interventions either 1) vehicle (10% DMSO in NSS), 2) deferoxamine (25 mg/kg), or 3) Mdivi‐1 (2 mg/kg). The control rats were also given a vehicle. All rats were received the assigned treatment for further 2 weeks. Behavioral tests including open field test (locomotor activities) and the Morris water maze test (spatial memory test) were evaluated. At the end of experiments, rats were euthanized, and serum samples were collected to assess serum iron level. Furthermore, brain tissues were preserved for the assessment of blood‐brain tight junction protein, brain iron level, and the protein expression related to brain mitochondrial fission and brain mitophagy.

**Results:**

Iron dextran‐injected rats demonstrated an increase in serum/brain iron levels, called as “iron‐overloaded condition”, blood‐brain barrier disruption, and increased brain mitochondrial fission and brain mitophagy, resulting in a decline in spatial memory. The administration of deferoxamine alleviated brain pathologies and partially mitigated the impaired spatial memory test in iron‐treated rats. Interestingly, the Mdivi‐1 exhibited greater neuroprotective effects than deferoxamine by attenuating brain mitophagy and restoring spatial memory **(Figure 1)**.

**Conclusion:**

Mdivi‐1 potentially exerted neuroprotective effects and cognitive restoration in iron‐overloaded rats, suggesting a novel therapeutic strategy using mitochondrial fission inhibitor in the brain following iron‐overloaded condition.